# Cluster of differentiation-44 as a novel biomarker of lupus nephritis and its role in kidney inflammation and fibrosis

**DOI:** 10.3389/fimmu.2024.1443153

**Published:** 2024-10-01

**Authors:** Caleb C.Y. Wong, Lucy Y. Gao, Yuesong Xu, Mel K.M. Chau, Danting Zhang, Desmond Y.H. Yap, Shirley K.Y. Ying, Cheuk Kwong Lee, Susan Yung, Tak Mao Chan

**Affiliations:** ^1^ Department of Medicine, School of Clinical Medicine, The University of Hong Kong, Hong Kong, Hong Kong SAR, China; ^2^ Department of Medicine and Geriatrics, Princess Margaret Hospital, Hong Kong, Hong Kong SAR, China; ^3^ Hong Kong Red Cross Blood Transfusion Service, Hong Kong, Hong Kong SAR, China

**Keywords:** lupus nephritis, CD44, kidney inflammation, tubulo-interstitial fibrosis, biomarker

## Abstract

**Introduction:**

CD44 is a transmembrane glycoprotein implicated in tissue inflammation and fibrosis. We investigated its role in kidney inflammation and fibrosis in a murine model of lupus nephritis (LN), and the clinico-pathological association of serum CD44 level in patients with biopsy-proven Class III/IV ± V LN.

**Methods:**

NZB/W F1 mice were treated with control IgG or anti-CD44 monoclonal antibody for 4 weeks and disease parameters assessed. Serum CD44 level in LN patients was determined by ELISA. Control groups included healthy subjects and patients with non-renal SLE or non-lupus renal disease.

**Results:**

CD44 expression was absent in the normal kidney, but it was expressed in proximal and distal tubular epithelial cells and infiltrating cells in renal biopsies from patients with active proliferative LN. ScRNA-Seq datasets confirmed that CD44 was predominantly expressed in tubular cells and all immune cells identified in LN patients including tissue resident, inflammatory and phagocytic macrophages, Treg cells, effector and central memory CD4^+^ T cells, resident memory CD8^+^ T cells and naïve and activated B cells. Treatment of NZB/W F1 mice with anti-CD44 antibody preserved kidney histology and reduced proteinuria, tubulo-interstitial infiltration of CD3^+^, CD4^+^ and CD19^+^ immune cells, and mediators of kidney fibrosis compared to Control mice. Longitudinal studies showed that serum CD44 level increased prior to clinical renal flare by 4.5 months and the level decreased after treatment. ROC curve analysis showed that CD44 level distinguished patients with active LN from healthy subjects and patients with quiescent LN, active non-renal lupus, and non-lupus CKD (ROC AUC of 0.99, 0.96, 0.99 and 0.99 respectively). CD44 level correlated with leukocyte infiltration and interstitial inflammation scores in active LN kidney biopsies.

**Discussion:**

Our findings suggest that CD44 plays a pathogenic role in renal parenchymal inflammation and fibrosis in active LN and monitoring CD44 may facilitate early diagnosis of flare.

## 1 Introduction

Lupus nephritis (LN) is a common and severe manifestation of systemic lupus erythematosus (SLE), and is an important cause of acute kidney injury and chronic kidney disease (CKD) (1). LN is initiated by the deposition of immune complexes in the glomerular and tubular basement membranes triggering complement activation, proliferation of resident renal cells, recruitment of immune cells and induction of inflammatory and fibrotic processes, which if not adequately controlled, will lead to scarring and loss of renal reserve (1, 2). Reducing acute immune-mediated kidney damage and preventing kidney fibrosis is essential to ensure optimum long-term kidney and patient survival. Kidney biopsy remains the gold standard for confirming the diagnosis of LN and subsequent assessment of histopathological changes in the kidney, but since it is invasive it cannot be performed frequently. Conventional investigations such as lupus serological tests to assess anti-dsDNA and C3 levels reflect immunological status but not organ injury, and their change over time is useful in many but not all patients (3, 4). Indicators of kidney injury or function such as proteinuria and serum creatinine are insensitive and could present late in the course of disease activation, and they may not accurately reflect histopathological changes (5, 6). There is a pressing need to identify novel molecules that not only serve as diagnostic and prognostic biomarkers but contribute to LN pathogenesis and serve as potential therapeutic targets.

CD44 is a transmembrane glycoprotein widely expressed on the surface of both immune and non-immune cells including monocytes, lymphocytes, epithelial cells and fibroblasts. CD44 plays important roles in cell migration, proliferation, cell-matrix interaction, and presentation of cytokines including TGF-β1, to their cognate receptors thereby inducing downstream tissue fibrosis. CD44 is a major cell surface receptor for hyaluronan (HA) and CD44-HA interactions have been reported to induce murine B cell activation and effector functions in T cells and macrophages (7, 8). The ability of CD44 to bind HA is dependent on its activation status, which can be induced by pro-inflammatory mediators including TNF-α, IL-1β, and RANTES (9, 10). CD44 is also a component of the endothelial glycocalyx, a delicate gel-like layer located at the interface between the endothelium and bloodstream, and it impedes leukocyte binding to cell adhesion molecules and their transmigration from the circulation across the endothelium to sites of injury (11). Acute or chronic inflammation induces endothelial cell activation, which results in the shedding of glycocalyx constituents and their detection in the circulation (12), and exposes cell adhesion molecules that medicate immune cell infiltration to sites of injury.

In the normal kidney, CD44 expression is restricted to passenger leukocytes and resident macrophages and is weakly expressed in the mesangium (13–15). Renal expression of CD44 is markedly increased in experimental models of kidney injury, and human nephropathies such as IgA nephropathy, crescentic glomerulonephritis and membranoproliferative glomerulonephritis (14–16). In patients with IgA nephropathy, renal CD44 expression strongly correlated with the degree of glomerular and tubulo-interstitial damage, and proteinuria (17). In lupus-prone MRL-*lpr* mice, increased CD44 expression was observed in perivascular inflammatory infiltrates, glomerular crescents and cortical tubules (15). The role of CD44 in LN pathogenesis has not been defined.

This study investigated the role of CD44 in kidney inflammation and fibrosis in NZB/W F1 mice and its potential role as a novel biomarker in the diagnosis and clinical management of LN.

## 2 Materials and methods

### 2.1 Chemicals, reagents and assay kits

All chemicals were of the highest purity and were purchased from Sigma Aldrich (Tin Hang Technology, Hong Kong) unless otherwise stated. Human CD44 and mouse VCAM-1 Duoset ELISA kits were purchased from R&D Systems Inc. (Genetimes Technology International Holding Limited, Hong Kong). Mouse CD44 ELISA kits were purchased from Lifespan Biosciences, Inc (Bio-Gene Technology Limited, Hong Kong). Kallestad™ anti-dsDNA microplate EIA kits and DC Protein Assay kits were purchased from Bio-Rad Pacific Limited, Hong Kong. QuantiChrom™ Urea, Creatinine and Albumin Assay Kits were purchased from BioAssay Systems (California, USA). Tissue culture flasks were purchased from Falcon (Becton Dickinson, Gene Company Limited, Hong Kong), and L-glutamine were purchased from Life Technologies (Thermo Fisher Scientific, Hong Kong). Rat B cell hybridomas that produce monoclonal antibody against mouse CD44 (clone IM7.8.1) were purchased from American Type Culture Collection (ATCC, Tin Hang Technology Limited, Hong Kong). EX-CELL hybridoma medium^®^, Hematoxylin solution (Gill No. 3), Eosin Y solution, Bouin’s solution, Biebrich Scarlet-Acid Fuchsin Solution and Weigert’s Iron Hematoxylin solution were purchased from Sigma-Aldrich (Tin Hang Technology Limited, Hong Kong). HiTrap^®^ Protein G High Performance column was purchased from GE Healthcare (Tin Hang Technology, Hong Kong). Pierce™ High Capacity Endotoxin Removal Spin Columns, IgG2b Rat Uncoated ELISA kits, Taqman^®^ gene expression assay probes for mouse CD44 (Mm01277163_m1), VCAM-1 (Mm01320970_m1), ICAM-1 (Mm00516023_m1), TGF-β1 (Mm00441726_m1), α-smooth muscle actin (α-SMA) (Mm00725412_s1) and fibronectin (FN) (Mm00692666_m1), and mouse GAPDH endogenous control (VICTM/MGB probe, primer limited) were purchased from Thermo Fisher Scientific, Hong Kong Limited. RNeasy mini kits were purchased from Qiagen Hong Kong Pte Limited, Hong Kong. Non-immune rat anti-mouse IgG was purchased from MP Biomedicals (Genetimes ExCell Technology Inc. Hong Kong).

### 2.2 Preparation of mouse anti-CD44 antibody

Rat B cell hybridomas (clone IM7.8.1) were cultured in EX-CELL hybridoma medium supplemented with L-glutamine (10mM, final concentration) and the medium changed every 2-3 days. Endotoxin-free anti-CD44 monoclonal antibody was purified from the culture medium by protein G-Sepharose chromatography using HiTrap^®^ Protein G High Performance columns followed by Pierce™ High Capacity Endotoxin Removal Spin Columns. The purity of the antibody was assessed by SDS-PAGE (**Supplementary Figure 1A**), and IgG concentration determined using IgG2b Rat Uncoated ELISA kits according to the manufacturer’s instructions. Anti-CD44 antibody was filtered sterilized using 0.22 μm filter, stored at -20°C in endotoxin-free PBS at a concentration of 1mg/ml until required.

### 2.3 Animal studies

Female NZB/W F1 mice were bred from female NZB and male NZW mice purchased from the Jackson Laboratory (Bar Harbor, Maine, USA) under an AAALAC International accredited program at the Centre for Comparative Medicine Research, the University of Hong Kong under specific pathogen free conditions. Mice were kept under normal housing conditions in a 12-hour night and day cycle, and water and chow were available *ad libitum*. Treatment started when mice were 25 - 28 weeks of age when they developed proteinuria, defined as spot albumin-to-creatinine ratio (ACR) greater than 10 mg/g on two occasions at least 2 days apart. In the first study, NZB/W F1 mice were randomized into 2 groups to receive either non-immune rat anti-mouse IgG (Control) or anti-CD44 antibody (CD44 Ab) (10 μg), once weekly by intravenous administration for 4 weeks, after which time mice were sacrificed, blood collected, and kidneys harvested (n = 4 - 6 mice per group). This dose represented the lowest dose of CD44 antibody that could reduce proteinuria after 4 weeks compared to control IgG (**Supplementary Figure 1B**). In a separate study, female NZB/W F1 mice were randomized into 2 groups to receive either vehicle or mycophenolate mofetil (MMF, 100 mg/kg/day), once daily by oral gavage for 4 weeks, and blood and kidneys harvested. The MMF dose used was previously shown to reduce inflammation and fibrosis in NZB/W F1 mice (18–20). Animal studies were reviewed and approved by the Committee on the Use of Live Animals in Teaching and Research (CULATR) at the University of Hong Kong.

### 2.4 Renal histopathology

Paraffin-embedded kidney sections (5 μm) from NZB/W F1 mice treated with either Control IgG or anti-CD44 antibody were stained with H&E and Masson’s trichome as previous described (19). Renal histology was scored by two independent observers in a blinded manner. Briefly, following H & E staining, kidney lesions in the glomerular and tubulo-interstitial compartments were graded 0 to 3 (0 = normal, 1 = mild, 2 = moderate and 3 = severe) and expressed as mean glomerular and tubulo-interstitial lesion scores for each group (19). For each mouse, approximately 20 glomeruli, tubular, interstitial and vascular areas were evaluated for glomerular hypercellularity, mesangial matrix expansion, crescent formation, influx of mononuclear cells, fibrinoid necrosis, hyaline deposits, tubular atrophy, protein cast deposition and vasculopathy (18, 21, 22). For semi-quantitative assessment of Masson’s trichrome staining, collagen-positive area was assessed using computer-assisted image analysis software (ImageJ, NIH, USA).

### 2.5 Cytochemical staining

In NZB/W F1 mice, paraffin-embedded kidney sections (5 μm) were stained for CD44, CD3, CD4, CD19, F4/80, hyaluronan binding protein (HABR), VCAM-1 and NGAL as previously described (23). Signal detection was by the peroxidase method, visualized by 3,3’-diaminobenzidine (DAB) and counterstained with Haematoxylin. CD44 expression was assessed in paraffin-embedded normal kidney specimens (5 μm) from patients undergoing nephrectomy (n = 6) and renal biopsies from patients with active proliferative LN (n = 15) using cytochemical staining as previously described (23). Signal detection and visualization was by the peroxidase-anti-peroxidase method and specimens were counterstained with Haematoxylin. Five to ten non-overlapping images were taken for each specimen using a Zeiss™ Axioscope 5 upright microscope and Axiocam 208 digital camera system (Carl Zeiss Far East Company Limited, Hong Kong), and semi-quantitative assessment of mediators of inflammation and fibrosis was performed using ImageJ software (NIH, USA).

### 2.6 Gene expression

Total mRNA was extracted from the kidney cortex of NZB/W F1 mice using RNeasy mini kits according to the manufacturer’s instructions. Two micrograms of total mRNA were reverse-transcribed into cDNA with Primescript™ RT reagent kit with gDNA Eraser, and gene transcripts for CD44, VCAM-1, ICAM-1, TGF-β1, α-SMA and FN assessed by quantitative real-time PCR using Taqman gene expression assay on a Lightcycler 480 II real time PCR system (Roche Diagnostics, DKSH Hong Kong Limited, Hong Kong). All samples were analyzed in triplicate, and mRNA expression of the aforementioned markers of inflammation and fibrosis were calculated using the delta delta Ct (2−ΔΔCt) method, normalized to GAPDH.

### 2.7 Patients and controls

Archived sera collected during the period February 2000 to August 2015 from 41 adult Chinese patients with kidney biopsy-proven Class III/IV ± V LN under active follow-up at the SLE clinic at Queen Mary Hospital, Hong Kong were included in this study. Paired sera, one collected during renal flare and the other during quiescent, were obtained from 39 patients and were used in the comparison between active disease and remission, and also in the cross-sectional study. In addition, serial sera collected from 39 patients at intervals of 3 to 4 months during long-term follow-up with at least one sample collected during active disease, were used in the longitudinal study. Patients with serum creatinine above 450 µmol/l and eGFR below 15ml/min were excluded since renal function deterioration *per se* is associated with altered immune responsiveness (24), and could confound the results. Disease activity was classified as ‘active’ or ‘inactive’ based on clinical and serological parameters of disease such as proteinuria, active urinary sediment, with or without deteriorating kidney function (25). All renal flares were confirmed with kidney biopsy, whereas inactive renal disease was defined by insignificant proteinuria and stable kidney function in patients on stable low-dose maintenance immunosuppressive treatment, typically at a daily dose of prednisolone ≤5 mg. Renal SLEDAI-2K was calculated as previously described (25–27). Corresponding clinical, serological, and histopathological parameters were retrieved from database and hospital records. Standard therapy for active LN was prednisolone and mycophenolate mofetil (MMF), at tapering doses, as both initial and maintenance therapy continuously (28). Activity and Chronicity Indices in renal biopsies were determined as previously reported, with maximum scores of 24 and 12 respectively (27). Sera from age- and sex-matched healthy subjects (n = 46), patients with non-proliferative CKD and comparable eGFR as LN patients (n = 37), and SLE patients without renal involvement (n = 53), served as controls. The studies involving human participants were reviewed and approved by the University of Hong Kong and Hospital Authority Hong Kong West Cluster Institutional Review Board (HKU/HA HKW IRB), and all subjects gave written consent for the use of their serum and clinical data in this study.

### 2.8 ELISAs and assays

All samples were measured in duplicate. Anti-dsDNA antibody level in LN and SLE patients was measured using Kallestad™ anti-dsDNA microplate EIA kits according to the manufacturer’s instructions, with a detection range of 20 - 300 IU/ml. Serum CD44 and VCAM-1 levels in patients and/or mice were measured using commercially available ELISAs according to the manufacturers’ instructions. The detection range for human CD44 Duoset ELISA kits was 78.10 - 5,000 pg/ml. Seropositivity was defined as values greater than mean + 3 SD in healthy subjects, with a cut-off value of 1.36 ng/ml.

In animal studies, mouse CD44 and VCAM-1 ELISA kits had detection range of 0.156 - 10 ng/ml and 125 - 8,000 pg/ml respectively. Serum creatinine and urea levels were measured using QuantiChrom™ Creatinine and Urea assay kits respectively. Spot urine was collected weekly from Control IgG-treated and anti-CD44 antibody-treated mice, and ACR was determined using QuantiChrom™ Albumin and Creatinine assay kits to assess disease progression.

### 2.9 Analysis of CD44 expression from public sequencing datasets

Published Bulk RNA-Seq transcriptomic datasets from NBCI Gene Expression Omnibus [GEO accession no. GSE127797 (29), GSE32591 (30), GSE69438 (31), and GSE37463 (32)], and single cell data of AMP Phase 1 (33, 34) were retrieved to assess CD44 expression in micro-dissected glomeruli and tubules from patients with biopsy-proven LN and healthy control subjects. The expression matrix for each GEO series was downloaded, and batch normalization of microarrays was performed using the R package limma and sva. ScRNA-Seq datasets from resident renal cells (33) and immune cells (34) from renal specimens from LN patients and healthy controls were used to assess CD44 in individual cell populations using Seurat R package (v 5.0.1). Highly expressed ubiquitous gene, including mitochondrially encoded and nuclear-genome encoded ribosomal proteins, were excluded from clustering and removed. For the single cell dataset comprising resident renal cells and immune cells, highly variable genes were loaded to a PCA analysis utilizing the top principal components for clustering and t-SNE visualization. Cell annotation for tubular cells and immune cells was conducted as described (33, 34).

### 2.10 Construction of protein-protein interaction network

The protein-protein interaction (PPI) network was constructed by mapping CD44 and mediators of inflammation and fibrosis identified in our animal study to the Search Tool for the Retrieval of Interacting Genes (STRING) (35), with the full STRING network having an interaction score set to 0.7 and false discovery rate (FDR) set at <0.05. Biological processes and molecular functions from Gene Ontology, KEGG Pathways and Disease-gene associations were retrieved.

### 2.11 Statistical analyses

All data were presented as mean ± SEM unless otherwise stated. Statistical analysis was performed using GraphPad Prism 10.1.0 for Windows (GraphPad Software, California, USA) and SPSS version 26 (SPSS, Chicago, Illinois, USA). Normal distribution was assessed using the D’Agostino-Pearson normality test. Differences were assessed by Wilcoxon signed-ranked test for nonparametric paired data, or Mann-Whitney test for nonparametric unpaired data. For intra-group and inter-group comparisons with three groups or more, Kruskal-Wallis test followed by Dunn’s multiple comparison post-test was used for nonparametric data. Mouse survival rate was determined using Fisher’s exact test. Correlation between CD44 and serological and clinical markers of disease was examined using nonparametric Spearman’s correlation coefficient. Fisher’s Exact Test was used to compare categorical variables between patients with or without LN. The sensitivity and specificity of serum CD44, anti-dsDNA antibody and C3 levels, proteinuria and renal SLEDAI-2K score in distinguishing patients with active LN from different comparator groups was assessed using receiver operating characteristic (ROC) curve analysis. Two-tailed P<0.05 was considered statistically significant.

## 3 Results

### 3.1 Renal CD44 expression in patients and mice with active LN

**Figure 1:**
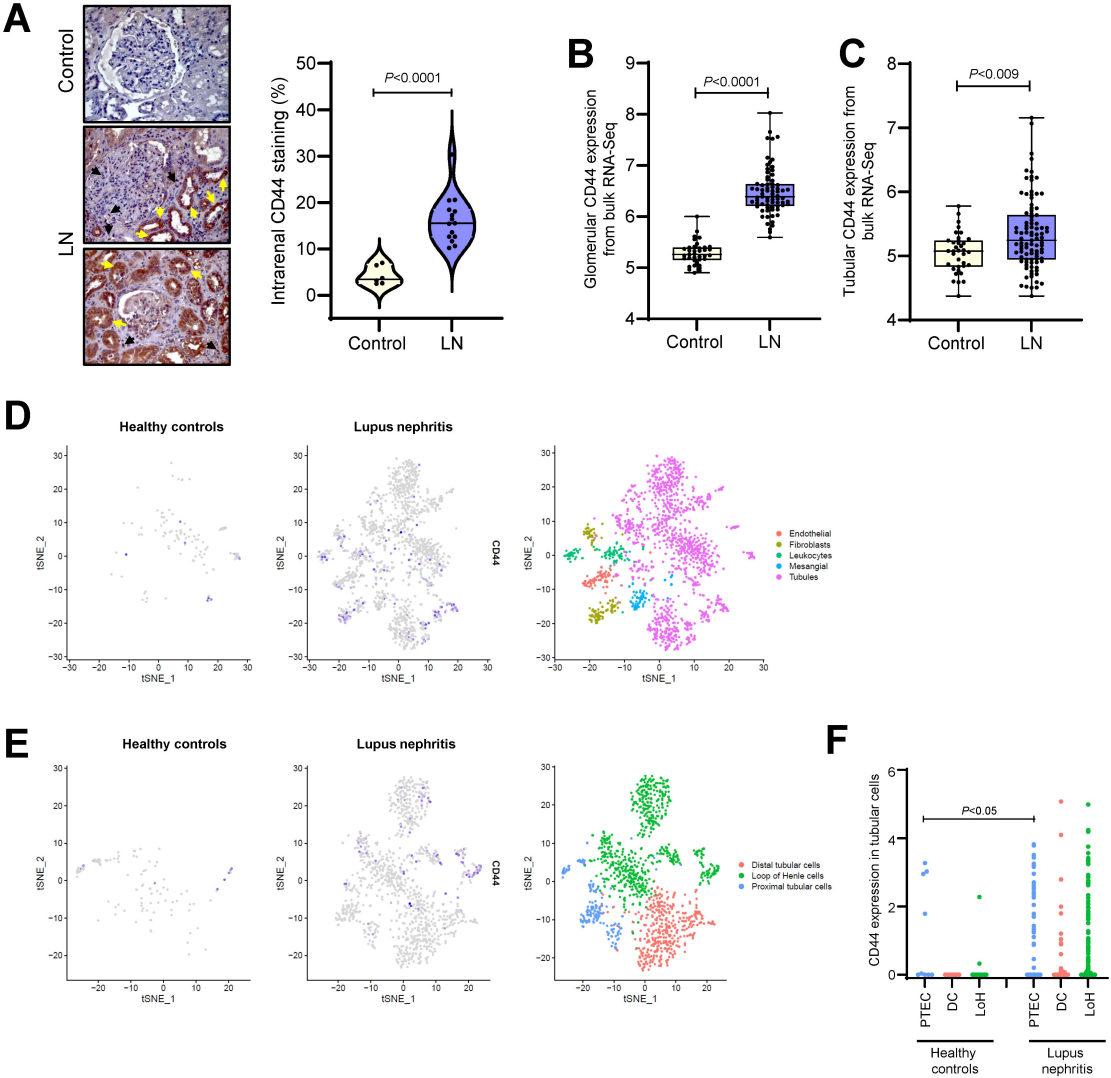
CD44 expression in renal specimens from LN patients. **(A)** Left panel: representative images of CD44 staining in normal kidney specimens (Control, n = 6) and patients with active proliferative LN (n=15). Black arrows depict recruitment of CD44^+^ immune cells into the kidney and yellow arrows indicate CD44 staining in tubular cells. Original magnification x400. Right panel: violin plot showing CD44-positive staining as a percentage of the whole image area using ImageJ software. Each dot represents CD44 staining from an individual control or LN patient. Data analysed using Mann-Whitney test. Box and Whiskers plots comparing CD44 gene expression in micro-dissected **(B)** glomeruli from 37 healthy controls and 73 LN patients and **(C)** tubulo-interstitial compartments from 36 healthy controls and 95 LN patients. Data obtained from bulk RNA-Seq datasets GSE127797, GSE32591, GSE69438 and GSE37463. **(D)** tSNE plots showing CD44 expression in resident renal cells using public scRNA-Seq dataset obtained from 21 LN patients and 3 healthy controls (33). Each dot represents a single cell and grey to blue colour represents low to high CD44 expression. **(E)** tSNE plots showing CD44 expression in renal tubular cells (n = 1223 cells) after sub-clustering according to their canonical markers: *ALDOB* and *MIOX* for PTEC (n = 177 cells), *CALB1* for distal tubular cells (DC, n = 400 cells) and *UMOD* and *SLC12A1* for Loop of Henle cells (LoH, n = 646 cells). Data obtained from public scRNA-Seq dataset from 21 LN patients and 3 healthy controls (33). **(F)** Histogram showing CD44 expression in renal tubular cells after sub-clustering in healthy controls and LN patients.

**Figure 2:**
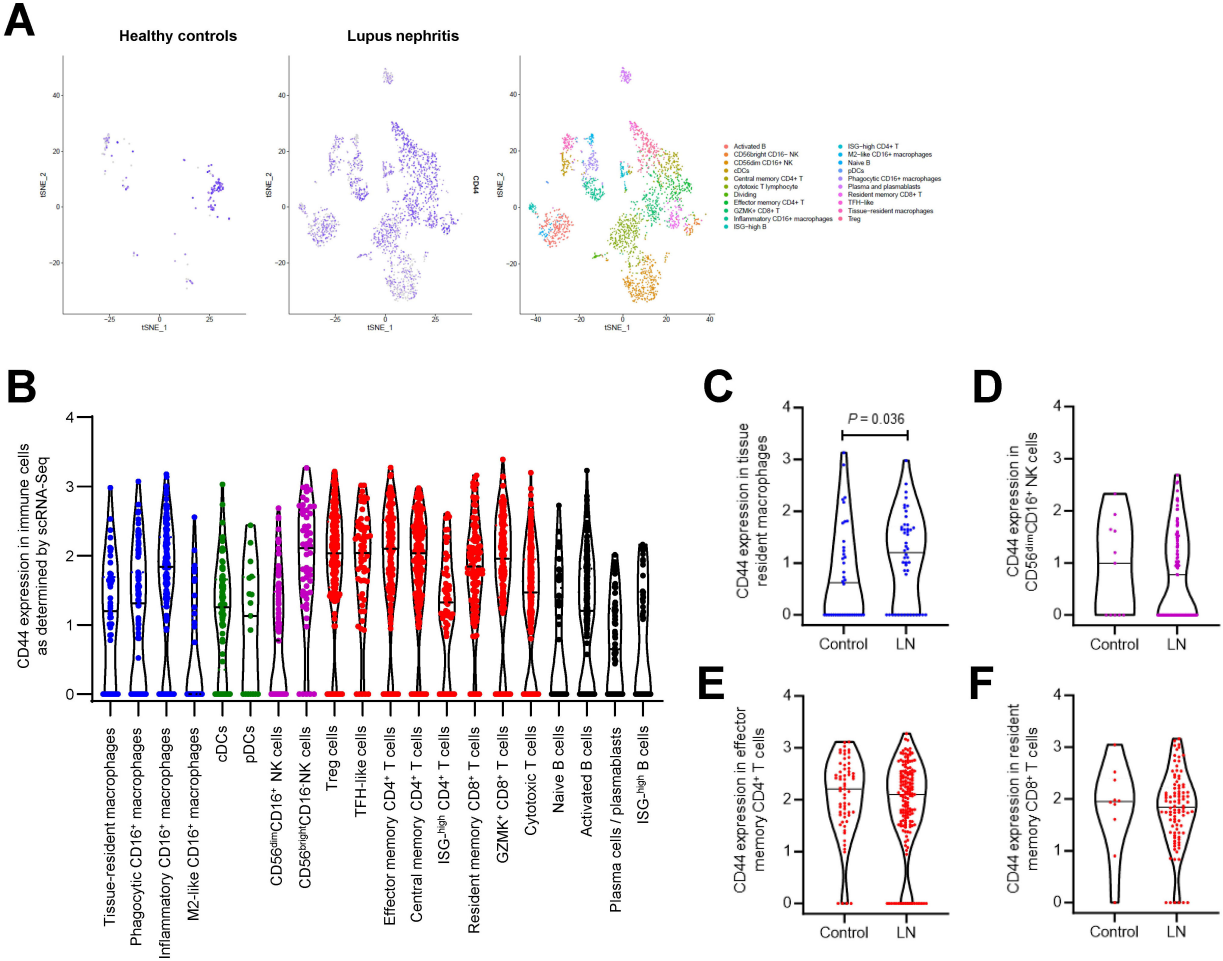
CD44 expression in immune cells in LN patients. **(A)** tSNE plots showing CD44 gene expression in immune cells using public scRNA-Seq dataset obtained from kidney specimens from 24 LN patients and 10 healthy controls (34). **(B)** Violin plots showing CD44 gene expression in different subsets of macrophages, dendritic cells, T cells and B cells in LN patients. Violin plots showing CD44 expression in **(C)** tissue resident macrophages **(D)** CD56^dim^CD16^+^ NK cells, **(E)** effector memory CD4^+^ T cells and **(F)** resident memory CD8^+^ T cells in 10 healthy controls and 24 LN patients. Each dot represents a single cell. Horizontal line represents mean value for each group.

CD44 expression was absent in the normal kidney, but it was expressed in tubular cells and infiltrating immune cells in renal biopsies from patients with active proliferative LN (**Figure 1A**). We utilized public bulk and single-cell RNA-Seq datasets obtained from micro-dissected glomeruli and tubulo-interstitial compartments, resident renal cells (33) or immune cells (34) to confirm our findings. CD44 gene expression was significantly increased in glomeruli and within the tubulo-interstitium in LN patients compared to control samples (**Figure 1B, C**), and was predominantly expressed in tubular cells attributed to PTEC, distal tubular cells and cells in the Loop of Henle (**Figures 1D–F**). In healthy subjects, CD44 was expressed on resident macrophages, CD56^dim^CD16^+^NK cells, effector memory CD4^+^ T cells and resident memory CD8^+^ T cells, whereas CD44 was expressed in all immune cells identified in LN patients including tissue resident, inflammatory and phagocytic macrophages, naïve and activated B cells, Treg cells, effector and central memory CD4^+^ T cells and resident memory CD8^+^ T cells (**Figures 2A–F**). Of the immune cells identified in both healthy subjects and LN patients, CD44 expression in tissue resident macrophages was significantly increased in LN patients compared to healthy subjects (**Figure 2C**).

**Figure 3:**
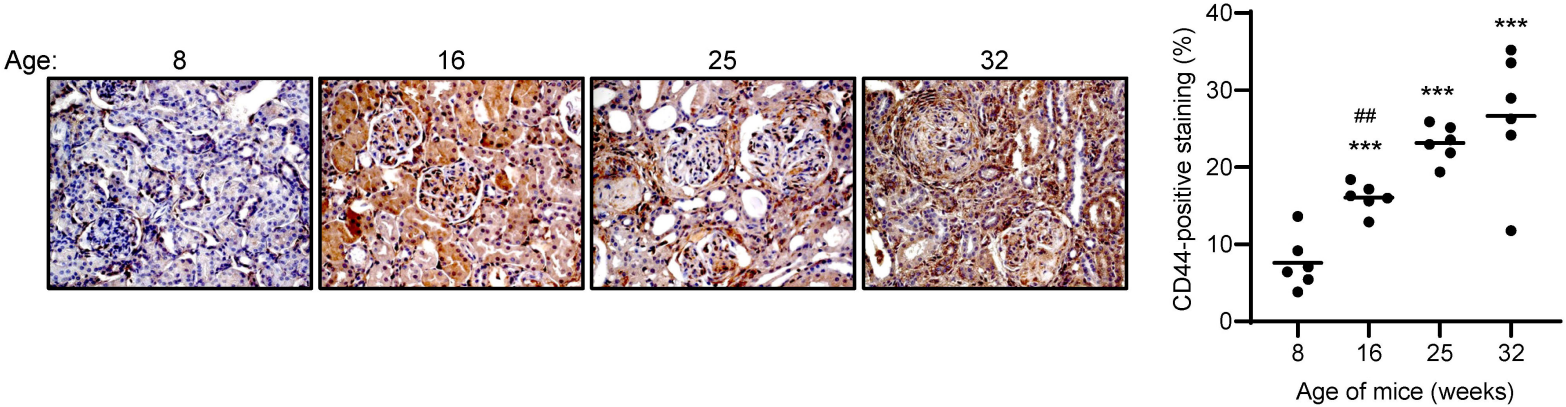
CD44 expression in NZB/W F1 mice with progressive LN. Left panel: Representative images showing CD44 expression in NZB/W F1 mice with progressive disease, where 8, 16, 25 and 32 weeks of age represent different stages of disease, namely pre-nephritis, emergence of anti-dsDNA antibodies, active nephritis and severe disease respectively (n = 6 mice per timepoint). Original magnification x200. Right panel: scatterplot showing CD44-positive staining as a percentage of the whole image area using ImageJ software. Each dot represents an individual mouse. Horizontal line represents the mean for each group. ***P<0.001, compared to 8 weeks of age, ^##^ *P*<0.001, 16 weeks vs 32 weeks. Data analysed using Kruskal-Wallis test followed by Dunn’s multiple comparison post-test.

Renal biopsies provide a snap-shot of histopathological changes at the time of biopsy, whereas longitudinal animal studies allow researchers to monitor histopathological changes with time. In NZB/W F1 mice, CD44 expression was negligible in 8-week old pre-nephritic mice, and detected in PTEC and glomeruli, localized to mesangial cells, endothelial cells, and infiltrating immune cells, at the time of anti-dsDNA antibody emergence at 16 weeks of age. As disease progressed from acute kidney injury to CKD, CD44 was detected in crescents, areas of interstitial fibrosis, and on infiltrating immune cells in both the glomerular and tubulo-interstitial compartments suggesting that LN pathogenesis is accompanied by an increase in CD44 expression (**Figure 3**).

### 3.2 Effect of anti-CD44 antibody on LN pathogenesis

**Figure 4:**
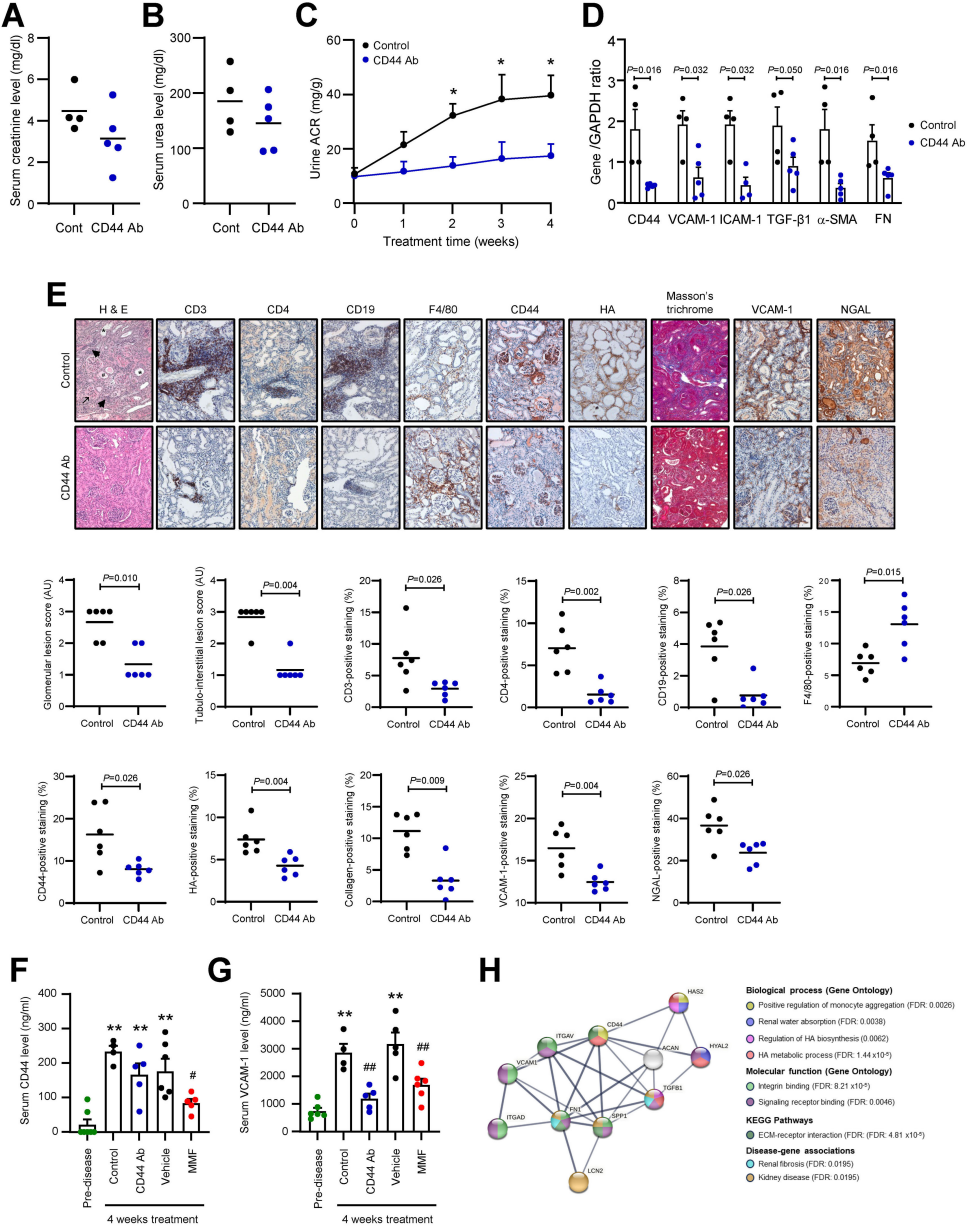
Effect of anti-CD44 antibody on clinical, serological and histological parameters of disease in NZB/W F1 Mice. Serum **(A)** creatinine and **(B)** urea levels in NZB/W F1 mice treated with either Control IgG (Cont, n = 4) or anti-CD44 antibody (CD44 Ab, n = 5) for 4 weeks. **(C)** Urine albumin-to-creatinine ratio (ACR) determined weekly from commencement of study to study’s end for mice treated with Control IgG (n = 4) or anti-CD44 antibody (n = 5). *P<0.05, Control IgG vs anti-CD44 antibody for the same time-point. **(D)** Gene expression of CD44, VCAM-1, ICAM-1, TGF-β1, α-SMA and FN in the renal cortex from NZB/W F1 mice treated with either Control IgG (Control, n = 4) or CD44 Ab (n = 5) for 4 weeks. Each sample was assessed in triplicate by qPCR, normalized to GAPDH and each dot represents the mean value for each mouse. Data analysed using Mann-Whitney test for each gene. **(E)** Upper panel: representative images showing H & E, CD3, CD4, CD19, F4/80, CD44, HA, collagen (determined by Masson’s trichrome), VCAM-1 and NGAL staining in mice treated with either Control IgG (Control, n = 6) or CD44 Ab (n = 6) for 4 weeks. In H & E image, asterisks depict tubular atrophy, hashtag depicts protein cast formation, arrowhead depicts immune cell infiltration and arrow depicts areas of glomerulosclerosis. Original magnification x200. Lower panels: Glomerular and tubulo-interstitial lesion scores as determined by H & E staining was graded for each mouse as described in the Methods and Materials. Scatterplots showing staining of CD3^+^ T cells, CD4^+^ T cells, CD19^+^ B cells and macrophages as determined by F4/80 staining, and expression of CD44, HA, collagen, VCAM-1 and NGAL as a percentage of the whole image area as assessed by ImageJ software. Horizontal line represents the mean for each group. Data analysed using Mann-Whitney test. **(F)** Serum CD44 and **(G)** Serum VCAM-1 levels in NZB/W F1 mice treated with Control IgG (n = 4) or CD44 Ab (n = 5) for 4 weeks. In a parallel study, serum CD44 and VCAM-1 levels were measured in mice treated with vehicle (n = 5) or MMF (n = 6) for 4 weeks. Serum from 8-week old pre-disease mice (n = 6) served as baseline CD44 and VCAM-1 levels. **P<0.01, compared to pre-disease mice, ^#^ *P*<0.05, ^##^ *P*<0.01, Control vs CD44 Ab, or Vehicle vs MMF. Each dot represents an individual mouse. Data analysed using Kruskal-Wallis test followed by Dunn’s multiple comparison post-test. Mice were 29-32 weeks of age at the time of sacrifice for panels **(A–G)**. Although 6 mice were assigned to Control IgG- and anti-CD44 antibody-treated groups, frozen biological samples from 2 Control IgG-treated mice and 1 anti-CD44 antibody-treated mouse were compromised, and these 3 samples were not used for subsequent clinical **(C)**, serological **(A, B, F, G)** or mRNA analyses **(D)**. **(H)** PPI network for CD44, HA, FN (FN1), TGF-β1, VCAM-1 and NGAL (LCN2) constructed using STRING database, with a minimum required interaction score of 0.7 (high confidence). Eleven nodes were identified with 27 edges (PPI enrichment *P* value 5.56 x10^-10^). Line thickness indicates confidence level of protein-protein interaction. Coloured nodes show their interaction in various biological processes, molecular functions, KEGG pathways and association with disease. False discovery rates (FDR) are shown. ACAN, aggrecan; HAS2, hyaluronan synthase 2; HYAL2, hyaluronidase-2; ITGAD, integrin αD (receptor for VCAM-1); ITGAV, integrin αV; SPP1, osteopontin (ligand for CD44).

We next investigated whether suppressing CD44 activation using neutralizing anti-CD44 antibody could improve disease manifestation in NZB/W F1 mice. All mice survived after 4 weeks’ treatment with either Control IgG or anti-CD44 antibody. Nephritis in the kidney of Control IgG-treated mice was accompanied by anti-dsDNA antibody production, proteinuria and detection of serum creatinine and urea levels (**Figures 4A–C**). Histopathological changes in the kidney included glomerular hypertrophy, glomerulosclerosis, tubular dilation, protein cast formation and pronounced infiltration of mononuclear cells in the periglomerular and tubulo-interstitial area (glomerular lesion score: 2.67 +/- 0.52 AU; tubulo-interstitial lesion score: 2.83 +/- 0.41 AU), attributed in part by CD3^+^ and CD4^+^ T cells, CD19^+^ B cells and macrophages. CD44 was predominantly expressed in the glomeruli and localized in the mesangium, Bowman’s capsule and crescents. CD44 was also expressed in the tubulo-interstitium and in infiltrating cells although to a lesser extent. Administration of anti-CD44 antibody to mice significantly reduced CD44 expression at the transcription and translation level, and was accompanied by preservation of the kidney structure, and a significant decrease in proteinuria, and CD3^+^ and CD4^+^ T cell and CD19^+^ B cell infiltration, and an increase in macrophages in the tubulo-interstitium. Mediators of inflammation and fibrosis including HA, TGF-β1, α-SMA, FN, collagen, VCAM-1 and ICAM-1 were also significantly decreased at the gene and/or protein level in CD44 Ab-treated mice (**Figures 4D, E**). Neutrophil gelatinase-associated lipocalin (NGAL), also known as lipocalin-2 (LCN-2), a marker of acute tubular injury, was expressed in dilated proximal tubules in Control mice, and its expression was significantly reduced in CD44 Ab-treated mice (**Figure 4E**).

Serum VCAM-1 and CD44 levels were significantly increased in Control IgG-treated mice compared to pre-disease mice. Serum VCAM-1, but not CD44 level, was significantly reduced in CD44 Ab-treated mice compared to Control mice (**Figures 4F, G**). We previously demonstrated that MMF improved serological, clinical and histological parameters of disease in NZB/W F1 mice (18–20). After 4 weeks’ treatment, MMF significantly reduced serum CD44 and VCAM-1 levels to pre-nephritic levels compared to vehicle-treated mice (**Figures 4F, G**).

Using STRING, we assessed the putative interaction of CD44 with 5 markers of fibrosis and tubular injury including HA, VCAM-1, TGF-β1, FN, and NGAL identified in our animal studies. These 6 proteins were connected by 11 nodes and 27 edges and demonstrated a significant PPI network (PPI enrichment *P* value = 5.56x10^-10^). From the network, CD44 may induce HA synthesis and tubular injury indicated by NGAL/LCN2 expression through increased HAS2 activity and FN expression respectively. CD44 may directly regulate biological processes such as monocyte aggregation and ECM-receptor interaction, and regulate renal water absorption, signaling and kidney disease/fibrosis through its interaction with HA, HAS2, hyaluronidase, TGF-β1 and FN (**Figure 4H**).

### 3.3 Clinico-pathological association of CD44 level in LN patients

In our animal studies, we demonstrated that serum CD44 level was increased in vehicle-treated mice compared to pre-nephritic mice, whereas CD44 level in MMF-treated mice was comparable to pre-disease level (**Figure 4F**). To determine whether serum CD44 level could serve as a potential biomarker for LN, we measured serum CD44 level in 490 serum samples from 41 LN patients. Demographics and clinical characteristics of LN patients are presented in **Table 1**. None of the patients had anti-phospholipid syndrome or antiphospholipid antibodies. Nine patients had newly diagnosed active LN in the inclusion period. Serum creatinine level was similar between LN patients with active disease and CKD controls (113.70 ± 64.66 vs 93.09 ± 29.31µmol/l, *P*=0.35). In the cross-sectional study, patients with active LN and remission had SLEDAI-2K score of 11.74 ± 2.87 (range 6 to 18) and 2.72 ± 2.72 (range 0 to 8) respectively. Extra-renal manifestations included arthritis (n=2), skin rash (n=1), pleurisy (n=1), fever (n=1), thrombocytopenia (n=5), leukopenia (n=3). Thirteen patients had sera collected before renal relapse.

**Table 1:** Demographics and clinical characteristics of LN patients in cross-sectional study.

	Renal flare	Remission	*P* value
Age (year)	37.74 ± 10.74	39.80 ± 10.37	<0.0001
Gender (F: M)	29: 12	27: 12	>0.999
^a^Duration of SLE (y)	5.08 (0.25, 15.57)	7.44 (3.29,17.94)	0.136
^a^Duration of LN (y)	4.81 (0.04, 13.32)	7.01 (2.87, 14.37)	0.079
SLEDAI-2K score	11.74 ± 2.87	2.72 ± 2.72	<0.0001
Renal SLEDAI-2K score	5.37 ± 3.18	0.61 ± 1.46	<0.0001
Serum anti-dsDNA antibody (IU/ml)	133.10 ± 100.30	80.44 ± 86.48	0.023
Serum C3 (mg/dl)	47.74 ± 18.10	80.33 ± 25.64	<0.0001
eGFR (ml/min/1.73m^2^)	68.84 ± 29.81	81.78 ± 29.14	0.036
Serum creatinine (µmol/l)	113.70 ± 64.66	106.70 ± 138.40	0.047
Serum urea (mmol/l)	12.12 ± 9.38	7.64 ± 5.76	0.002
Serum albumin (g/l)	29.29 ± 6.79	41.22 ± 3.19	<0.0001
Urine albumin-to-creatinine ratio (mg/mmol Cr)	470.00 ± 332.60	54.78 ± 35.20	<0.0001
Serum IgG (mg/dl)	1287.00 ± 811.30	1278.00 ± 464.20	0.569
Serum IgA (mg/dl)	270.00 ± 136.70	226.00 ± 91.92	0.375
Serum IgM (mg/dl)	89.77 ± 58.54	78.24 ± 46.20	0.599
Prednisolone dose (mg/day)	32.53 ± 12.85	6.81 ± 1.17	<0.0001
Renal biopsy activity score	7.14 ± 3.38		
Renal biopsy chronicity score	2.02 ± 2.03		
Correlation of serum CD44 level with activity score	0.37		0.025
Correlation of serum CD44 level with leukocyte infiltration	0.45		0.018
Correlation of serum CD44 level with interstitial inflammation	0.37		0.049

Results expressed as mean ± SD.

a Duration of SLE and LN expressed as median (25 and 75% percentile).

Fifty-three patients with non-renal SLE (46 females and 7 males, 47.96 ± 10.32 years of age, *P*=0.617 compared to LN patients) were included, among whom 18 had blood samples collected during active disease and 35 during remission, with SLEDAI-2K scores of 5.56 ± 3.45 (range 2 to 12) and 2.63 ± 2.49 (range 0 to 8) respectively (**Table 2**). Clinical manifestations in active non-renal SLE patients included arthritis (n=4), leukopenia (n=3), thrombocytopenia (n=7) and vasculitis (n=4). Patients with active non-renal SLE were treated with prednisolone alone (n=1), or prednisolone together with MMF (n=4), azathioprine (n=8), everolimus (n=1) and cyclosporin A (n=4).

**Table 2:** Demographics and clinical characteristics of non-renal SLE patients in cross-sectional study.

	Active non-renal SLE (n=18)	Non-renal SLE in remission (n=35)	*P* value
Age (year)	50.61 ± 9.00	46.60 ± 10.80	0.195
Gender (F: M)	15:3	31:4	0.678
^a^Duration of SLE (y)	20.00 (15.00, 26.00)	13.00 (7.00, 22.00)	0.031
SLEDAI-2K score	5.56 ± 3.45	2.63 ± 2.49	0.001
Serum anti-dsDNA antibody (IU/ml)	98.22 ± 72.96	57.68 ± 64.78	0.020
Serum C3 (mg/dl)	82.94 ± 22.00	81.94 ± 21.88	0.842
Prednisolone dose (mg/day)	14.17 ± 8.36	6.71 ± 3.81	<0.0001
Clinical manifestations during flare:			
Arthritis	4		
Vasculitis	4		
Thrombocytopenia	7		
Leukopenia	3		

Results expressed as mean ± SD.

a Duration of SLE expressed as median (25 and 75% percentile).

**Figure 5:**
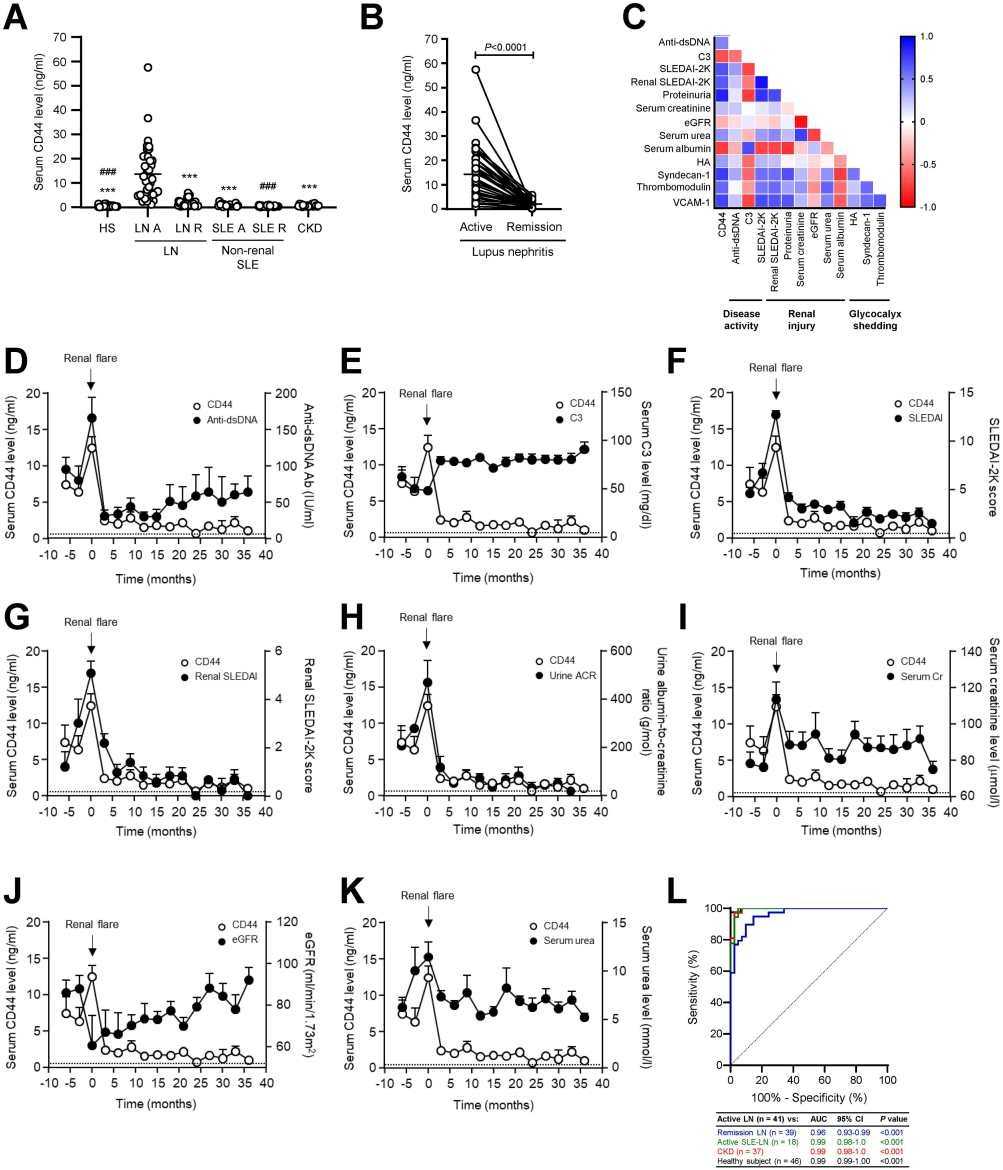
Serum CD44 level in LN patients and control groups. **(A)** Scatterplot showing serum CD44 level in healthy subjects (n = 46), LN patients with active disease (LN A, n = 41) or remission (LN R, n = 39), non-renal SLE patients with active disease (SLE A, n = 18), non-renal SLE patients in remission (SLE R, n = 35) and non-lupus CKD patients (n = 37). ***P<0.001, compared to LN A; ^###^ *P*<0.001, compared to LN R. Data analysed using Kruskal-Wallis test followed by Dunn’s multiple comparison post-test. **(B)** Serum CD44 level was determined in paired serum samples whereby one sample was obtained during active disease and another during remission (n = 39). Horizontal line represents mean for each group. Data analysed using Wilcoxon signed-ranked test. **(C)** Heatmap showing correlation of serum CD44 level with serological and clinical parameters of disease (n = 80). Graphs showing the temporal relationship between serial serum CD44 level and **(D)** anti-dsDNA antibody level, **(E)** serum C3 level, **(F)** SLEDAI-2K score, **(G)** renal SLEDAI-2K score **(H)** urine albumin-to-creatinine ratio, **(I)** serum creatinine level, **(J)** eGFR and **(K)** serum urea level in LN patients. Results are expressed as mean ± SEM (n = 39). Dashed line represents mean CD44 level in healthy subjects. **(L)** ROC AUC analysis of serum CD44 level in distinguishing patients with active LN (n = 41) from patients with quiescent LN (blue line, n = 39), active non-renal SLE (green line, n = 18), CKD (red line, n = 37) or healthy subjects (black line, n = 46).

Seropositivity rate for CD44 in LN patients with active disease and remission was 97.56% and 33.33% respectively compared to 70.37% and 42.31% respectively for anti-dsDNA antibody titre. Serum CD44 level was significantly increased in patients with active LN compared to quiescent LN patients, patients with active non-renal SLE, CKD patients or healthy subjects (13.58 ± 1.73, 1.57 ± 0.22, 0.57 ± 0.11, 0.67 ± 0.08 and 0.37 ± 0.05ng/ml respectively, *P*<0.001, active LN vs remission LN, non-renal active SLE, CKD and healthy subjects) (**Figure 5A**). Serum CD44 level was also significantly higher in quiescent LN patients compared to quiescent non-renal SLE patients and healthy subjects (1.57 ± 0.22, 0.54 ± 0.07, 0.37 ± 0.05 ng/ml, *P*<0.001, quiescent LN vs quiescent non-renal SLE patients or healthy subjects). Serum CD44 level was similar between LN patients in remission and CKD patients (*P*=0.143). In paired samples, all patients with active LN showed higher serum CD44 level compared to their corresponding remission sample (**Figure 5B**).

Serum CD44 level correlated with anti-dsDNA antibody level (r=0.49, *P*=0.0002), proteinuria (r=0.84, *P*<0.0001), serum creatinine (r=0.27, *P*=0.017) and serum urea (r=0.44, *P*=0.0005) levels, and both SLEDAI-2K (r=0.67, *P*<0.0001) and renal SLEDAI-2K (r=0.65, *P*<0.0001) scores. Serum CD44 level also correlated with serum levels of HA (r=0.40, *P*=0.013), syndecan-1 (r=0.75, *P*<0.0001), thrombomodulin (r=0.59, *P*<0.0001), and VCAM-1 (r=0.73, *P*<0.0001) levels. CD44 level showed an inverse correlation with C3 level (r=-0.69, *P*<0.0001), eGFR (r=-0.31, *P*=0.006), and serum albumin level (r=-0.76, *P*<0.0001). When compared to conventional markers of disease activity (anti-dsDNA antibody and C3 levels) and kidney damage (proteinuria, serum creatinine and renal SLEDAI-2K score), CD44 level showed a better correlation with eGFR, and comparable correlation with serological and clinical parameters of disease (**Figure 5C**). Serum CD44 level showed no association with patients’ age or duration of SLE or LN (data not shown).

Longitudinal studies included 39 LN patients. In 13 patients who had sera collected before active disease, CD44 level was increased in 10 patients (76.92%) at 4.50 ± 1.43 months prior to relapse and at the time of flare in the remaining 3 patients, whereas anti-dsDNA antibodies increased in 6 patients (46.15%) prior to clinical flare, in 1 patient at the time of flare and the remaining 6 patients showed normal anti-dsDNA antibody level during active LN. CD44 level decreased after treatment and returned to basal level approximately 9 months after commencing induction immunosuppressive treatment. A temporal relationship was observed between CD44 level and anti-dsDNA and C3 levels, SLEDAI-2K and renal SLEDAI-2K scores, proteinuria and eGFR (**Figures 5D–K**). At the time of flare, 1 out of 39 patients showed normal CD44 level but high anti-dsDNA antibody titre, whereas 12 out of 39 patients showed normal anti-dsDNA antibody level.

ROC analysis showed that serum CD44 level distinguished active LN from healthy subjects with sensitivity and specificity rates of 97.56% and 100.00% respectively (*P*<0.001), from LN patients in remission with sensitivity and specificity rates of 89.74% and 90.24% respectively (*P*<0.001), from active non-renal SLE with sensitivity and specificity rates of 100.00% and 95.12% respectively (*P*<0.001), and from CKD patients with sensitivity and specificity rates of 100.00% and 97.56% respectively (*P*<0.001) (**Figure 5L**, **Table 3**). CD44 level showed a higher sensitivity rate than anti-dsDNA antibody and C3 levels in distinguishing between patients with active LN and LN remission with sensitivity and specificity rates of 73.08% and 55.56% respectively for anti-dsDNA antibody, and 74.36% and 84.21% respectively for C3 level (**Table 3**). CD44 showed comparable sensitivity and specificity rates as proteinuria (sensitivity and specificity rates of 88.89% and 100.00% respectively) and renal SLEDAI-2K scores (sensitivity and specificity rates of 84.62% and 87.80% respectively) in distinguishing between patients with active LN and those in remission. Furthermore, CD44 level also showed comparable sensitivity and higher specificity than serum creatinine (sensitivity and specificity rates of 84.62% and 35.00% respectively) in distinguishing active LN and remission. CD44 level showed higher sensitivity and specificity than anti-dsDNA titre and C3 level in distinguishing between active LN and active non-renal SLE (sensitivity and specificity rates of 71.43% and 70.37% respectively for anti-dsDNA antibodies, and 85.71% and 86.84% respectively for C3) (**Table 3**).

**Table 3:** Sensitivity and specificity rates of serum CD44 level and conventional markers in distinguishing patients with active LN from healthy subjects and patients with quiescent LN, non-renal SLE or non-lupus renal disease.

Biomarker	Comparison	Sensitivity (%)	Specificity (%)	AUC	*P* value	Cut off
**CD44**	**Active LN vs:**					
	Healthy subjects	97.56	100.00	0.99	<0.001	>1.86
	LN patients in remission	89.74	90.24	0.96	<0.001	>3.33
	Active non-renal SLE	100.00	95.12	0.99	<0.001	>2.41
	CKD control	100.00	97.56	0.99	<0.001	>1.93
**Anti-dsDNA Ab**	LN patients in remission	73.08	55.56	0.68	0.02	>89.50
	Active non-renal SLE	71.43	70.37	0.72	0.07	>74.10
**C3**	LN patients in remission	74.36	84.21	0.89	<0.001	<64.00
	Active non-renal SLE	85.71	86.84	0.91	<0.001	<67.00
**SLEDAI-2K score**	LN patients in remission	87.18	87.80	0.90	<0.001	>5.50
	Active non-renal SLE	81.82	75.61	0.80	0.003	>7.50
**Renal SLEDAI-2K score**	LN patients in remission	84.62	87.80	0.89	<0.001	>2.00
**Urine albumin-to-creatinine ratio**	LN patients in remission	88.89	100.00	0.98	<0.001	>89.50
**Serum creatinine**	LN patients in remission	84.62	35.00	0.62	0.05	>114.00

Studies of histopathological association showed that at the time of active disease, serum CD44 level correlated with activity index (r=0.37, *P*=0.025), leukocyte infiltration score (r=0.45, *P*=0.018) and interstitial inflammation (r=0.37, *P*=0.049) in corresponding kidney biopsies, but not with the other components in activity or chronicity indices. Anti-dsDNA antibody and C3 levels and renal SLEDAI-2K score did not correlate with components in activity or chronicity indices, whereas proteinuria correlated with leukocyte infiltration score (r=0.52, *P*=0.018), and serum creatinine with interstitial inflammation (r=0.46, *P*= 0.037).

## 4 Discussion

This study aimed to delineate the role of CD44 in LN pathogenesis and whether it could serve as a target of therapeutic intervention. We hypothesized that increased renal CD44 expression contributes to immune cell recruitment into the kidney parenchyma and inhibition of CD44 activation in NZB/W F1 mice, by systemic treatment with IM7.8.1 monoclonal anti-CD44 antibody would improve disease manifestation. This method of targeting CD44 has been used in murine models of collagen-induced arthritis and diabetes, and both showed improvement in disease pathogenesis (36–39). After 4 weeks treatment, glomerular and tubulo-interstitial CD44 expression was significantly reduced and this was accompanied by a significant reduction in CD3^+^, CD4^+^ T cell and CD19^+^ B cell infiltration into the tubulo-interstitium. Reduced CD44 expression was also accompanied by a decrease in proteinuria, interstitial HA expression and mediators of kidney inflammation and fibrosis compared to Control IgG-treated mice. Our study suggests a pathogenic role for CD44 in LN through its ability to recruit B and T cells into the kidney and induction of kidney inflammation and fibrosis leading to kidney function deterioration. Treatment of mice with anti-CD44 antibody reduced CD44 expression at the transcription level, which may reduce its biosynthesis. Shedding of endothelial glycocalyx constituents serves as an indicator of active LN (11, 40, 41). Serum CD44 and VCAM-1 levels were significantly increased in Control IgG-treated mice compared to pre-disease mice. Treatment of mice with anti-CD44 antibody significantly reduced serum VCAM-1 level compared to Control IgG-treated mice, whereas serum CD44 level was comparable to that in Control IgG-treated mice. This suggests that the reduction in renal CD44 expression in anti-CD44 antibody-treated mice may also be attributed to CD44 shedding into the circulation. In line with our observation, in a murine model of rheumatoid arthritis, treatment of mice with IM7.8.1 anti-CD44 antibody was accompanied by a significant increase in serum CD44 level compared to baseline level (Day 0) (39). We previously demonstrated that MMF, the standard-of-care treatment for LN could exert direct anti-inflammatory and anti-fibrotic effects on resident renal cells and preserved kidney structure (19, 20, 23). In this study, MMF reduced serum CD44 and VCAM-1 levels compared to vehicle-treated mice, further highlighting the putative role of serum CD44 as a potential biomarker for disease activity and treatment efficacy in human and mice with active LN. It is intriguing to observe an increase in macrophages within the tubulo-interstitium in anti-CD44 antibody-treated mice. Macrophages present in the kidney express FcγR and binds autoreactive IgG immune complexes, which exacerbates kidney inflammation (42). Although the macrophage subset was not further investigated, given that suppression of CD44 activity was associated with preservation of kidney histology and decreased expression of mediators of inflammation and fibrosis would suggest that these macrophages aide repair and resolution of kidney inflammation and fibrosis.

Although the International Society of Nephrology/Renal Pathology Society have focused mainly on the classification of glomerular lesions in LN, up to 70% of LN patients show tubulo-interstitial inflammation, which if not adequately treated, can lead to tubular atrophy and interstitial fibrosis resulting in end-stage kidney disease (43). In this study, we demonstrated that CD44 plays a key role in kidney fibrosis since treatment of mice with anti-CD44 antibody attenuated tubulo-interstitial fibrosis. CD44, through its interaction with HA has been shown to activate TGF-β1 signaling through increased TGF-βRI kinase activity leading to SMAD phosphorylation (44). Furthermore, TGF-β1 can induce CD44 phosphorylation (44), suggesting an amplification loop. It is possible that anti-CD44 antibodies mediated its beneficial effect through suppression of CD44-HA interaction and subsequent TGF-β1 activation. Using the STRING database of known and predicted protein-protein interaction, CD44 showed interaction with TGF-β1. The STRING database also identified an interaction between CD44 and HAS2, one of three enzymes that regulate HA biosynthesis. We previously reported that anti-dsDNA antibodies increased HA synthesis in human mesangial cells through induction of HAS2 expression (45). A similar mechanism may also exist in renal tubular epithelial cells, although further studies are warranted to confirm this. Increased CD44 expression was associated with tubular injury in Control mice as demonstrated by increased expression of NGAL especially in tubules showing atrophy, whereas anti-CD44 antibody treatment reduced NGAL expression. Tubular atrophy is a predictor of CKD progression (46). Analysis of protein-protein interactions using STRING suggests that CD44 may induce NGAL through FN, which is deposited within the tubulo-interstitium during the early stages of tubulo-interstitial fibrosis (47), and serves as a scaffold for the deposition of other matrix proteins including collagen and laminin. In a murine model of obstructive nephropathy, CD44 deficiency was associated with a reduction in kidney fibrosis but increased tubular damage, the latter attributed to decreased proliferation and increased apoptosis of renal tubular epithelial cells, suggesting a protective effect of CD44 on tubular cells (48). The discrepancy between our findings and that of published results may be attributed to different murine models of kidney injury and mechanisms through which CD44 was depleted. We and others have reported that the ERK, mTOR and PI3K/AKT signaling pathways contribute to kidney inflammation, epithelial-to-mesenchymal transition and fibrosis (19, 20, 49, 50). Studies have shown that CD44 activation can activate ERK and PI3K/AKT signaling (51). It is possible that treatment of mice with anti-CD44 antibodies may reduce ERK and PI3K activation and downstream inflammation and fibrosis, and studies are ongoing to confirm this.

We next investigated the clinico-pathological association of serum CD44 level in LN patients. The purpose of this study was to determine whether measurement of CD44 could assist in clinical management by facilitating early diagnosis, and/or to provide a more sensitive and/or specific means of diagnosing active LN since conventional markers of kidney injury such as proteinuria could be due to factors other than disease activity, such as established glomerulosclerosis and hypertension. Our results showed increased serum CD44 level during active LN, and the level correlated with serological and clinical parameters of disease. Results from our longitudinal studies showed that serum CD44 level increased approximately 4.5 months before a nephritic flare was clinically evident and its level decreased after treatment in parallel with clinical improvement and returned to baseline after 9 months following induction therapy. This suggests that serial monitoring of serum CD44 level may serve as an early indicator of impending nephritic flare.

Immunological activity in LN is characterized by an increase in anti-dsDNA antibody titre and a decrease in C3 level. However, not all patients with active disease follow this characteristic serological profile, and it can be challenging to diagnose active nephritis in the early stage in these patients. Our study demonstrated that at the time of flare, 1 LN patient showed normal CD44 level, whereas 12 patients showed normal anti-dsDNA antibody level. The sensitivity and specificity rates for anti-dsDNA and C3 levels in distinguishing between active LN and quiescent disease in this study were similar to results published by independent investigators (52, 53), whereas CD44 was more sensitive and specific in distinguishing active LN from quiescent LN, active non-renal SLE and CKD, thereby demonstrating kidney specificity in SLE patients, and measuring serum CD44 level could assist in the diagnosis of active LN. Renal SLEDAI-2K score and proteinuria reflect kidney injury and are reliable markers of renal activity in active LN. CD44 showed comparative sensitivity and specificity with renal SLEDAI-2K score and urine ACR, and similar sensitivity and more specificity compared to serum creatinine level. When compared to conventional markers of immunological activity and kidney injury, serum CD44 level showed stronger correlation with serological and clinical parameters of active disease. The merits of our clinical study include a well-characterized patient group with biopsy-proven active severe LN with standard management protocols, cross-sectional studies and appropriate control groups comprising healthy subjects and patients with non-renal SLE or non-lupus CKD, and longitudinal follow-up data. However, a limitation of this study is the small number of patients, and a larger cohort of patients is necessary to validate our results.

Results from our histopathological studies showed that serum CD44 level was associated with activity index and correlated with the leukocyte infiltration and interstitial inflammation scores in renal biopsies from patients with active LN, whereas proteinuria and serum creatinine level was associated with one of these activity indices, and renal SLEDAI-2K score, anti-dsDNA antibody and C3 levels were not associated with parameters included in activity or chronicity indices. This may be attributed to marked inter-patient variation and highlights the importance of serial monitoring rather than relying on a single reading in informing clinical decisions. Class III/IV ± V LN is characterized by active and/or chronic lesions in glomeruli (54), and over 80% of kidney biopsies assessed in this study showed endocapillary hypercellularity and subendothelial hyaline deposits. That serum CD44 level showed no association with components of histologic activity indices relating to glomerular injury may be attributed to our observation that CD44 was predominantly expressed in renal tubular cells and may contribute to the development of tubulo-interstitial rather than glomerular lesions in active LN. Cytochemical staining of renal biopsies obtained at the time of active LN, showed that CD44 was predominantly expressed in tubules. It is possible that serum CD44 level in active LN patients may be attributed to shedding not only from the endothelial glycocalyx but also from renal tubular cells.

Transcriptomic analysis confirmed an increase in CD44 expression in kidney specimens from LN patients compared to healthy subjects, predominantly attributed to increased expression in renal tubular cells, and to a lesser extent in fibroblasts, mesangial cells and endothelial cells. Molecular profiling of kidney specimens from serial biopsies from LN patients showed that CD44 transcript was one of the top genes upregulated in glomeruli that contributed to inflammation and matrix expansion (55). Its increased expression in the second biopsy of non-responders despite treatment suggests its potential use as a marker of persistent glomerular inflammation and possible development of chronic renal damage (55). Since collection of renal biopsies is an invasive procedure, repeat biopsies are rarely performed. Animal models of LN allow the collection of serial kidney specimens to assess changes in CD44 expression with progressive disease. As in normal kidney specimens, CD44 expression was negligible in pre-nephritic NZB/W F1 mice. Immunological activity, characterized by the production of anti-dsDNA antibodies, was accompanied by induction of CD44 expression in both glomerular and tubular cells, and onset of nephritis and progression to CKD was accompanied by a significant increase in CD44 expression in crescents and within the tubulo-interstitium, the latter attributed in part by infiltration of CD44^+^ immune cells. Changes in the localization of CD44 in resident renal cells may thus depend on different stages of disease. Homing of activated T cells to kidney lesions is mediated in part through the binding of CD44 on the cell surface of T cells to HA in the endothelial glycocalyx (56). In healthy subjects, CD44 expression was localized to tissue resident macrophages, resident memory CD8^+^ T cells, effector memory CD4^+^ T cells and CD56^dim^CD16^+^ NK cells, whereas CD44 was expressed in all myeloid cells and lymphocytes in LN patients. These immune cells exert immunoregulatory functions through secretion of various pro-inflammatory cytokines or autoantibody production and can exacerbate inflammatory processes in the kidney. Studies have shown that increased expression of CD44 and variant isoforms CD44v3 and CD44v6 in CD4^+^ and CD8^+^ T cells endow them with an enhanced capacity to infiltrate the kidney and promote inflammation in SLE (57). Overexpression of CD44 in B cells is associated with B cell activation and may also promote their infiltration into the kidney (58, 59). Our animal studies demonstrated that suppression of CD44 activation can attenuate B and T cell infiltration into the kidney and preserve kidney structure.

**Figure 6:**
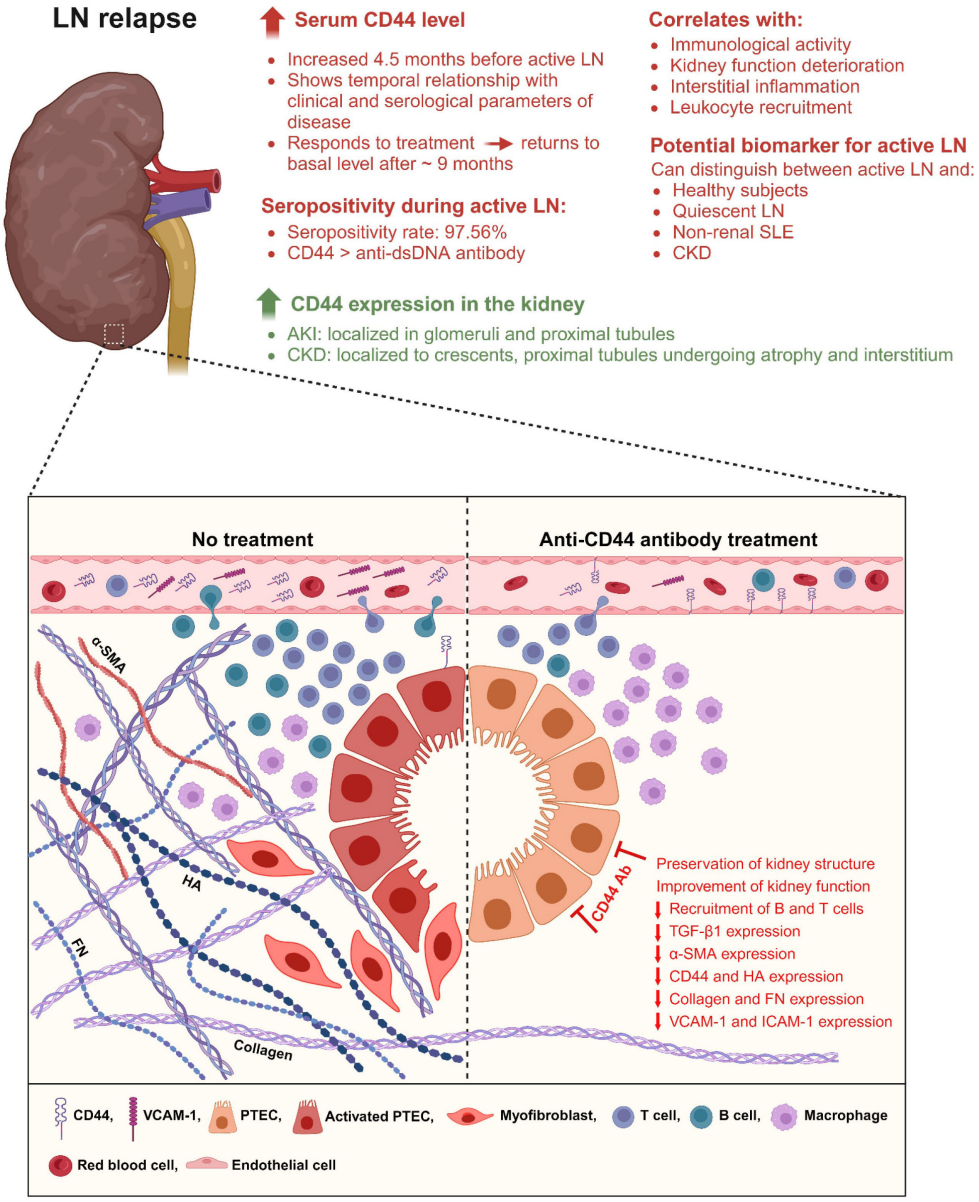
Schematic illustration showing the potential of CD44 as a novel biomarker of active LN, and its role in LN pathogenesis. During active LN, serum CD44 level is increased and correlates with serological and clinical parameters of disease. Its level is increased approximately 4.5 months before clinical symptoms of active LN is detected suggesting both predictive and prognostic potential. Serum CD44 level can distinguish between patients with active LN and quiescent LN, active non-renal SLE and non-lupus CKD suggesting specificity for renal manifestation in SLE patients. It is expressed by both immune and non-immune cells, and its location in the kidney parenchyma is dependent on disease progression, being present in glomerular and tubular cells and immune cells during increased immunological activity, and in crescents and interstitial space during onset of nephritis and kidney fibrosis. CD44 contributes to the recruitment of myeloid cells and lymphocytes into the tubulo-interstitium and increases tubulo-interstitial deposition of HA, α-SMA, collagen and FN resulting in tubular atrophy and proteinuria. Increased α-SMA expression may indicate epithelial-to-mesenchymal transition in PTEC. Treatment with anti-CD44 antibody attenuates histopathological changes in the kidney and reduces lymphocyte recruitment into the kidney parenchyma and this is associated with improvement in kidney function. CD44 may be a novel target of therapeutic intervention. Created with BioRender.com.

In conclusion, our findings highlight the pathogenic role of CD44 in mediating tubulo-interstitial inflammation and fibrosis in LN and the potential of CD44 as a therapeutic target. **Figure 6** summarizes our findings. Our study also demonstrated that serum CD44 level is increased in patients with active LN, and its level is associated with serological, clinical and histopathological parameters of disease, and it can distinguish between patients with active LN and remission. Collectively, these results suggest that measurement of CD44 level could facilitate early diagnosis of active LN, especially in patients in whom conventional serological markers do not appear helpful, and may also be useful in monitoring treatment response and histopathological changes in the kidney.

## Data Availability

The original contributions presented in the study are included in the article/**Supplementary Material**, further inquiries can be directed to the corresponding author/s.
