# Supplementary material for: Cluster of differentiation-44 as a novel biomarker of lupus nephritis and its role in kidney inflammation and fibrosis
Source: Front Immunol. 2024 Oct 1;15:1443153. doi: 10.3389/fimmu.2024.1443153 (PMC11473352; doi:10.3389/fimmu.2024.1443153)
Supplement: Supplementary file 1 [file DataSheet1.pdf]

Cluster of Differentiation-44 as a Novel Biomarker of Lupus Nephritis and Its Role in Kidney Inflammation and Fibrosis

Caleb CY Wong,[1†] Lucy Y Gao,[1†] Yuesong Xu,[1] Mel KM Chau,[1] Danting Zhang,[1] Desmond YH Yap,[1] Shirley KY Ying,[2] Cheuk Kwong Lee,[3] Susan Yung,[1*] Tak Mao Chan,[1*]

[1]Department of Medicine, School of Clinical Medicine, the University of Hong Kong, Hong Kong

[2]Department of Medicine and Geriatrics, Princess Margaret Hospital, Hong Kong

[3]Hong Kong Red Cross Blood Transfusion Service, Hong Kong

[†]These authors contributed equally to this work and share first authorship.

Supplementary Material

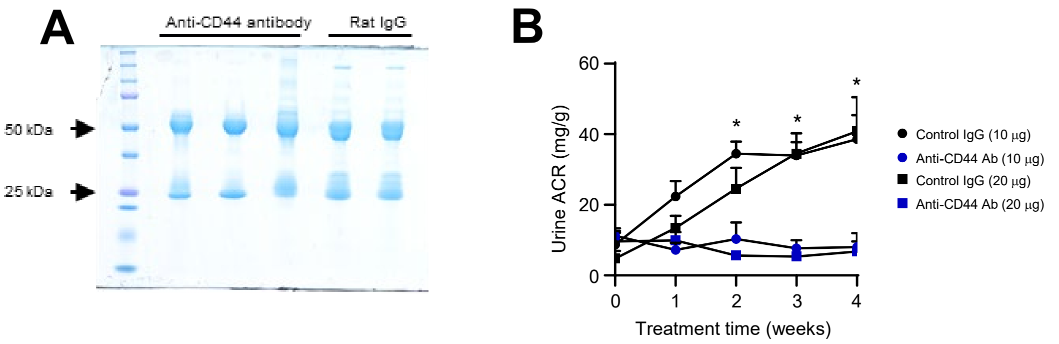

Supplementary Figure 1. Purification of anti-CD44 antibody and its effect on proteinuria

(A) Anti-CD44 monoclonal antibody was purified from the supernatant of rat B cell hybridomas (clone IM7.8.1) by protein G-Sepharose chromatography using HiTrap® Protein G High Performance columns followed by Pierce™ High Capacity Endotoxin Removal Spin Columns. The purity of the antibody was assessed by SDS-PAGE under reducing conditions using 30 μg total protein per lane. Purified IM7.8.1 antibody showed 2 bands at 25 kDa and 50 kDa representing the light and heavy chains of IgG respectively. BD Pharmingen™ purified NA/LE rat IgG2b,κ isotype control was also electrophoresed for comparison. (B) Preliminary studies showing the effect of weekly tail vein administration of Control IgG (10 or 20 μg, n = 4) or anti-CD44 antibody (10 or 20 μg, n = 5) on weekly urine ACR for 4 weeks (*P<0.05, compared to baseline (T = 0)).
